# Knockdown of astrocyte elevated gene-1 inhibits growth through suppression of IL-6 secretion in HepG2 human hepatoma cells

**DOI:** 10.3892/ol.2013.1645

**Published:** 2013-10-29

**Authors:** HUAN DENG, ZHENZHEN ZHOU, WEI TU, YUJIA XIA, HUANJUN HUANG, DE’AN TIAN

**Affiliations:** Department of Gastroenterology, Tongji Hospital, Tongji Medical College, Huazhong University of Science and Technology, Wuhan, Hubei 430030 P.R. China

**Keywords:** astrocyte elevated gene-1, proliferation, apoptosis, IL-6, HepG2 cells

## Abstract

Astrocyte-elevated gene-1 (AEG-1) has been reported to be associated with cancer progression in various types of human cancers, including liver cancer. However, to date, the molecular mechanism of AEG-1 action on the growth of liver cancer cells has been poorly elucidated. The present study aimed to investigate the effect of AEG-1 on the proliferation and apoptosis of liver cancer cells and the role of IL-6 in this process using the HepG2 human hepatoma cell line. shRNAs targeting AEG-1 were used to silence the expression of AEG-1. The effects on cell growth were detected by 3-(4, 5-dimethylthiazol-2-yl)-2, 5-diphenyltetrazolium bromide, colony formation and cell cycle assays. Apoptosis was analyzed by flow cytometry. The expression of IL-6 was examined by quantitative polymerase chain reaction and enzyme-linked immunosorbent assay, and the phosphorylation of Stat3 was detected by western blotting. AEG-1 knockdown was observed to induce cell proliferation inhibition and apoptosis through the suppression of IL-6 secretion. Stat3, a downstream target of IL-6 signaling, was suppressed in the AEG-1-silenced cells and target genes, including Bcl-2 and crystalin, αB, which are associated with cell survival, were downregulated. Overall, the findings suggest that aberrant AEG-1 expression promotes the growth of HepG2 liver cancer cells, contributing to the progression of liver cancer, which may partly be mediated by IL-6 signaling.

## Introduction

Liver cancer is one of the five most common types of cancer and a main cause for the mortality of patients with cancer (1). Liver cancer involves a rapidly growing tumor with a poor prognosis (2). However, the effects of the main therapeutic methods, including liver transplantation and surgical resection, remain limited (3). Therefore, the investigation of tumor-related genes may provide benefits for new treatment options.

Astrocyte elevated gene-1 (AEG-1), also named MTDH or LYRIC, was first cloned as an HIV- and TNF-α-inducible gene in primary human fetal astrocytes (9). Studies have suggested that AEG-1 expression was remarkably higher in glioma, breast, prostate, esophagus and liver cancers compared with that in the respective normal tissues (4–8). Previous studies have shown that AEG-1 synergizes with oncogenic Ha-ras to enhance the soft agar colony formation of non-tumorigenic immortalized melanocytes (10). Knockdown of AEG-1 induces apoptosis of prostate cancer cells through the downregulation of Akt activity and the upregulation of forkhead box 3a activity (6). In addition, AEG-1 may promote the progression of hepatocellular carcinoma by activating Wnt/β-catenin signaling (7). The data suggest that AEG-1 is able to induce growth promotion in liver cancer, but the molecular mechanism remains to be elucidated.

Gene silencing by RNA interference (RNAi) has become a powerful tool for functional genetic analyses due to the ability to create specific loss-of-function phenotypes. In the present study, RNAi was used to obtain the liver cancer HepG2 cell line with stably silenced expression of AEG-1. The effects of AEG-1 on proliferation and apoptosis were examined in the HepG2 cells. IL-6 is a significant inflammatory cytokine and play a key role in growth promotion in several human cancers, including biliary tract epithelial cancers, prostate cancer and multiple myeloma (11–12). IL-6 levels in patients with liver cancer are markedly higher than those in normal individuals (13). Furthermore, the high serum level of IL-6 has been shown to be closely associated with the progression of liver cancer (14). Data have suggested that IL-6 may activate several signaling pathways, including phosphoinositide 3-kinase, JAK/STAT and p38 mitogen-activated protein kinase by autocrine or paracrine pathways (15). Stat3, a key downstream target of IL-6 signaling, promotes the expression of several genes that are associated with cell survival, leading to tumor cell proliferation, apoptosis inhibition and an increased metastatic potential. The present study aimed to investigate the effects of AEG-1 on the proliferation and apoptosis of HepG2 cells, and explored the molecular mechanism of tumor growth in liver cancer.

## Materials and methods

### Cell culture and AEG-1-knockdown cells

Human liver cancer Hep3B, HepG2, SMMC-7721, MHCC-97H, HCC-LM3 and SK-HEP-1 cell lines were cultured in Dulbecco’s modified Eagle Medium (DMEM) with 10% fetal calf serum (Invitrogen Gibco, Carlsbad, CA, USA) and incubated at 37°C with 5% CO_2_. Two shRNA oligonucleotide duplexes against the AEG-1 sequence (NM_178812) were synthesized by GeneChem (Shanghai, China) according to the study by Yoo *et al*(7). The AEG-1 shRNAs were inserted into the psilencer2.0 vector. The successful plasmid construction was verified by DNA sequencing. The psilencer2.0-shAEG-1-1 and −2 and the psilencer2.0 control vector, respectively, were transfected into semi-confluent HepG2 cells using Lipofectamine 2000 reagent (Invitrogen Gibco). After 24 h, the transfected cells were trypsinized and replated into six-well plates (1:10), then selected for 14 days with 600 μg/ml G418 to produce stable AEG-1-knockdown cell lines. A single colony of stable cells was selected for further culture and the concentration of G418 was subsequently reduced by half and maintained in cultivation.

### 3-(4,5-dimethylthiazol-2-yl)-2,5-diphenyltetrazolium bromide (MTT) assay and flow cytometry analysis

A total of 5×10^3^ cells per well were plated into a 96-well plate. The cell growth was determined using the MTT assay with a wavelength of 550 nm. For the apoptosis analyses, the cells were harvested and stained using a phycoerythrin-Annexin V apoptosis detection kit (BD Pharmingen, San Diego, CA, USA) according to the manufacturer’s instructions. For the cell cycle analyses, the cells were harvested, washed twice with phosphate-buffered saline and fixed with precooled 75% ethanol. Prior to the flow cytometry analyses, the ethanol was removed and 500 μl freshly made dying solution, containing 0.05 mg/ml propidium iodide and 0.025 mg/ml RNase, was added. Subsequent to being stained for 30 min, the cells were subjected to flow cytometry analysis. A total of three replicates were performed.

### Colony formation assay

The HepG2-shAEG-1 and HepG2-vector cells were trypsinized and replated into six-well plates at a density of 5×10^2^ cells per well. After two weeks, the cells were washed twice with PBS and fixed with methanol/acetic acid (3:1; v:v) for 15 min. The fixing solution was removed and the cells were stained with 0.2% crystal violet for 10 min. The number of colonies was counted under a microscope (CKX41; Olympus, Tokyo, Japan).

### Quantitative polymerase chain reaction (qPCR)

Total RNA was isolated and extracted from the cells using TRIzol reagent according to the manufacturer’s instructions (Invitrogen). Total RNA (0.5 μg) was used for cDNA synthesis with PrimeScript RT reagent kit (Takara, Dalian, China). qPCR, based on SYBR premix EX TAQ (Takara), was performed to quantify the mRNA levels on an ABI StepOne Real-Time PCR system (Applied Biosystems, Inc., Carlsbad, CA, USA). The value of 2^−ΔΔCt^ was used to determine the fold changes between the samples. The sequences of the primers that were used in this study were as follows: Forward, 5′-CCTTGGGTCCA GTTGCCTTCT-3′ and reverse, 5′-CCAGTGCCTCTTTGCTG CTTTC-3′ for IL-6; forward, 5′-CATGTGTGTGGAGAGCG TCCA-3′ and reverse, 5′-GCCGGTTCAGGTACTCAGTCA-3′ for Bcl-2; forward, 5′-TCGGAGAGCACCTGTTGGA-3′ and reverse, 5′-CCATGTTCATCCTGGCGCTC-3′ for crystalin, αB (Cryab); and forward, 5′-GTTGCGTTACACCCTTTC TTG-3′ and reverse, 5′-GACTGCTGTCACCTTCACCGT-3′ for β-actin.

### Western blot analysis

For the western blot analysis, the cells were lysed in RIPA buffer (Sigma, St Louis, MO, USA). The protein concentration was determined using a BCA Protein Assay kit (Pierce, Rockford, IL, USA). Total protein (50 μg) was separated on a 10% SDS-PAGE gel and transferred to polyvinylidene difluoride membranes (Millipore, Bedford, MA, USA). The membranes were subsequently immunoblotted with primary antibody. Anti-AEG-1 antibody was purchased from Abcam (Cambridge, MA, USA) and anti-glyceraldehyde 3-phosphate dehydrogenase (GAPDH) was obtained from Epitomics, Inc. (Burlingame, CA, USA). Anti-p-Stat3 and -Stat3 antibodies were purchased from Cell Signaling Technology (Boston, MA, USA). A secondary goat anti-rabbit antibody solution (Santa Cruz Biotechnology Inc., Santa Cruz, CA, USA) was finally used for detection with an ECL kit (Pierce, Rockford, IL, USA), according to the manufacturer’s instructions.

### Enzyme-linked immunosorbent assay (ELISA) analysis

Semi-confluent HepG2-shAEG-1 and HepG2-vector cells were cultured in serum-free DMEM. The supernatants were collected after 48 h, centrifuged at 210 × g for 5 min at 4°C to remove the cellular debris, and stored at −80°C until the analysis was performed by ELISA. Immunoreactive IL-6 was quantified using the ELISA kit (R&D Systems, Emeryville, CA, USA), according to basic laboratory instructions. Each data point represents readings from a minimum of four independent assays that were performed in triplicate.

### Animal tumor model and xenograft

Eight male BALB/c-nu/nu mice (weight, ~20 g; age, four weeks old) were purchased from the animal center of Tongji Medical College, Huazhong University of Science and Technology (Wuhan, China). A total of five million HepG2-shAEG-1 and HepG2-vector cells in 0.1 ml PBS each were injected subcutaneously into the right and left flank of each nude mouse. The length (L) and width (W) of the tumors were measured externally using a vernier caliper every week. The tumor volume was determined according to the equation: V=(LxW^2^)/2. The growth curve was drawn according to the change in tumor volume over time. The mice were sacrificed following after weeks, and the tumors were excised and analyzed by hematoxylin and eosin staining. All experiments were performed according to the guidelines of the local animal use and care committee.

### Statistical analysis

The SPSS 16.0 software package (SPSS, Inc., Chicago, IL, USA) was used for the statistical analysis and measurement data are presented as the mean ± standard deviation. The statistical significance of the differences was determined using Student’s t-test. P<0.05 was considered to indicate a statistically significant difference.

## Results

### Establishing AEG-1-knockdown liver cancer cell lines

**Figure 1:**
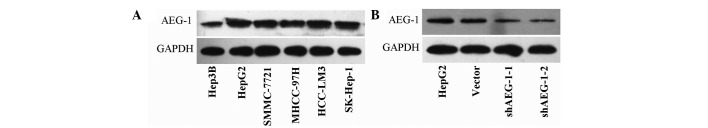
AEG-1 expression levels. (A) The expression of AEG-1 in six human liver cancer cell lines was analyzed by western blotting. (B) AEG-1 protein expression in AEG-1-silenced (HepG2-shAEG-1) and vector control (HepG2-vector) cells was detected by western blotting. GAPDH was used as a loading control. AEG-1, astrocyte-elevated gene-1; GAPDH, glyceraldehyde 3-phosphate dehydrogenase.

The protein expression of AEG-1 was assayed by western blotting in a panel of liver cancer Hep3B, HepG2, SMMC-7721, MHCC-97H, HCC-LM3, SK-Hep-1 cell lines. It was observed that the AEG-1 protein was overexpressed in all these liver cancer cell lines (Fig. 1A). The HepG2 cells exhibited high expression levels of AEG-1 and were selected for AEG-1 gene silencing. The psilencer2.0 (vector), psilencer2.0-shAEG-1-1 and 2 plasmids were stably transfected into the HepG2 cell line. Western blotting was used to detect the effect of AEG-1 silencing. Compared with the non-transfected HepG2 cells, the expression level of AEG-1 protein was remarkably inhibited in the HepG2-shAEG-1-1 and −2 cells. There was no significant difference between the HepG2-vector cells and the untreated HepG2 cells (Fig. 1B). The HepG2-shAEG-1-2 cells were chosen to complete the following experiments.

### Knockdown of AEG-1 inhibits cell growth and promotes apoptosis in hepatoma HepG2 cells

**Figure 2:**
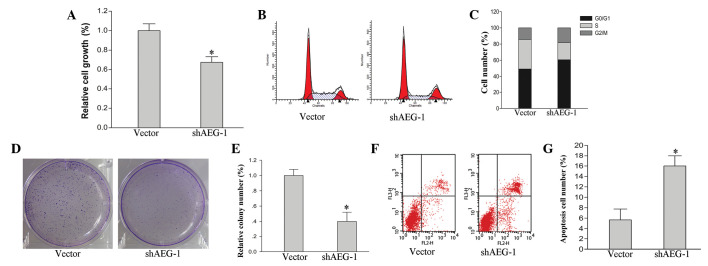
Effect of AEG-1 knockdown on the proliferation and apoptosis in HepG2 cells. (A) The proliferation of the AEG-1 knockdown and control cells was detected by MTT assay (^*^P<0.05). (B and C) FACS cell cycle analysis of AEG-1 knockdown cells and vector control. (D and E) Colony formation assay of the HepG2-shAEG-1 and HepG2-vector cells (^*^P<0.05). (F and G) Apoptosis analysis of the HepG2-shAEG-1 and HepG2-vector cells was detected by flow cytometry subsequent to staining the cells with Annexin V/PE (^*^P<0.05). Error bars represent SD, n=3 experiments. AEG-1, astrocyte-elevated gene-1; MTT, 3-(4, 5-dimethylthiazol-2-yl)-2, 5-diphenyltetrazolium bromide; FACS, fluorescence-activated cell sorting; PE, phycoerythrin.

To investigate the effects of AEG-1 on the growth of hepatoma HepG2 cells, cell cycle, cell proliferation and colony formation assays were performed. The cell cycle was significantly arrested in the HepG2-shAEG-1 cells. Compared with the vector control, the ratio of the cells in the G_0_/G_1_ phase was increased by 12% and the ratio of cells in the S phase was decreased by 16% in the HepG2-shAEG-1 cells (Fig. 2B and C). The cell proliferation ability was also inhibited by AEG-1 silencing. Knockdown of AEG-1 suppressed cell proliferation in the HepG2 cells by 26% (Fig. 2A; P<0.05). Furthermore, the ability of colony formation following AEG-1 silencing in the HepG2 cells was analyzed. The relative colony number was markedly decreased in the HepG2-shAEG-1 cells compared with the vector control (Fig. 2D and E; P<0.05). The knockdown of AEG-1-induced apoptosis by flow cytometry was examined in the HepG2 cells. Compared with the HepG2 cells that were transfected with empty vector plasmid, the apoptosis ratio increased significantly in the HepG2-shAEG-1 cells (Fig. 2F and G; P<0.05).

### Knockdown of AEG-1 suppresses IL-6 secretion and inhibits Stat3 activation

**Figure 3:**
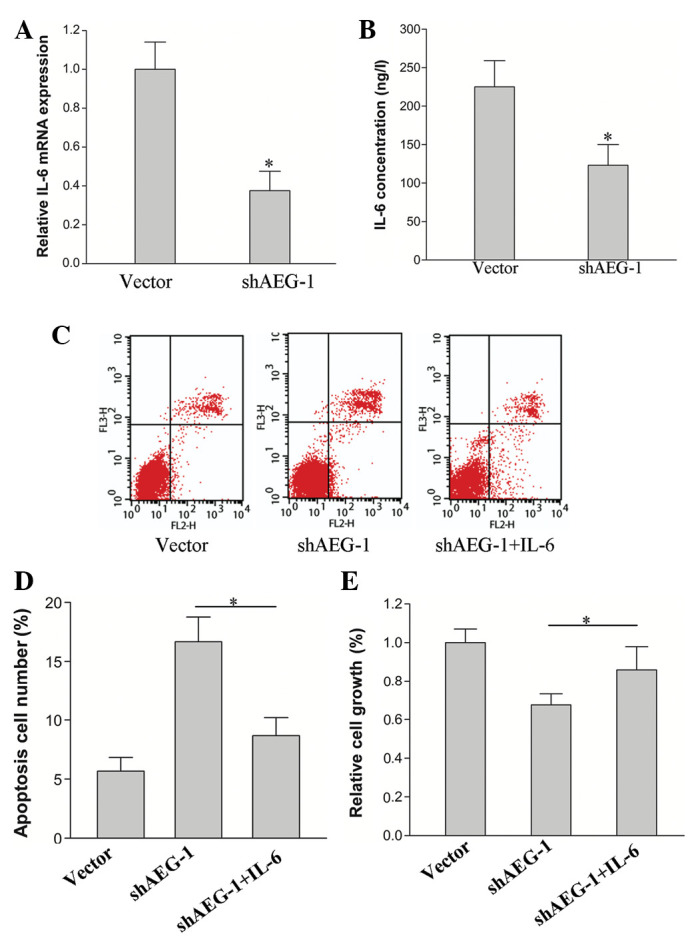
IL-6 expression in AEG-1 knockdown cells, and proliferation and apoptosis in HepG2-shAEG-1 cells treated with IL-6. (A and B) IL-6 expression was detected by qPCR and ELISA in the HepG2-shAEG-1 and HepG2-vector cells (^*^P<0.05). (C and D) Apoptosis analysis of the HepG2-shAEG-1 cells that were treated with IL-6 (50 ng/l) was assayed by flow cytometry (^*^P<0.05). (E) The proliferation of the HepG2-shAEG-1 cells that were treated with IL-6 (50 ng/l) or remained untreated was detected by MTT assay (^*^P<0.05). Error bars represent SD, n = 3 experiments. AEG-1, astrocyte-elevated gene-1; qPCR, quantitative polymerase chain reaction; ELISA, enzyme-linked immunosorbent assay; MTT, 3-(4, 5-dimethylthiazol-2-yl)-2, 5-diphenyltetrazolium bromide.

To determine whether IL-6 participates in the knockdown of AEG-1-induced growth inhibition and apoptosis, the expression of IL-6 in HepG2 cells was examined with AEG-1 silenced by shRNA. The mRNA level of IL-6 was decreased in the HepG2-shAEG-1 cells compared with that in the HepG2-vector cells (Fig. 3A; P<0.05). The concentration of IL-6 in the culture supernatants of the HepG2-shAEG-1 and HepG2-vector cells was identified to be similar to the expression levels of IL-6 mRNA. The secretion of IL-6 in the HepG2-shAEG-1 cells was reduced compared with that of the HepG2-vector cells (Fig. 3B; P<0.05). In the subsequent experiments, the proliferation and apoptosis of the HepG2-shAEG-1 cells was analyzed following the treatment with IL-6 (50 ng/l). The results revealed that the proliferation ratio increased in the HepG2-shAEG-1 cells that were treated with IL-6 compared with that in the untreated cells (Fig. 3E; P<0.05). The apoptosis ratio decreased in the IL-6-treated cells (Fig. 3C and D; P<0.05).

**Figure 4:**
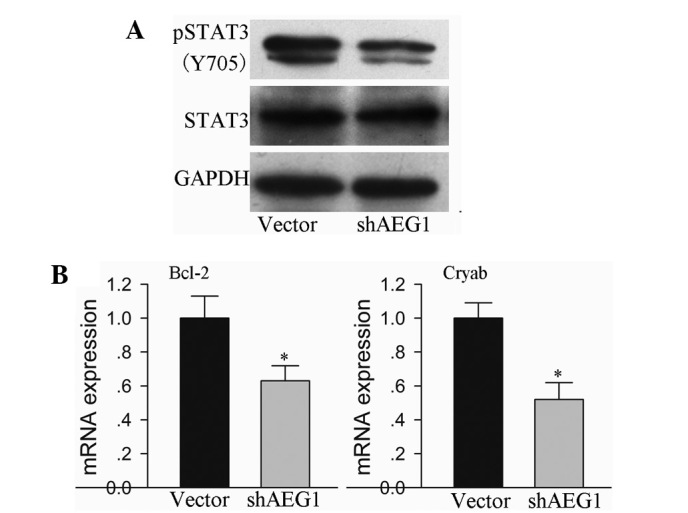
AEG-1 inhibition reduces activation of Stat3 signaling. (A) Stat3 expression levels were suppressed by AEG-1 silencing in HepG2 cells, as detected by western blotting. GAPDH was used as a loading control. (B) Stat3 target genes were downregulated in HepG2 cells with AEG-1 silencing, as detected by qPCR (^*^P<0.05). AEG-1, astrocyte-elevated gene-1; GAPDH, GAPDH, glyceraldehyde 3-phosphate dehydrogenase; qPCR, quantitative polymerase chain reaction; Cryab, crystalin, αB.

Stat3 is a significant transcription factor that is activated by IL-6 and associated cytokines. The present study demonstrated that AEG-1 inhibition was able to reduce the secretion of IL-6 in hepatoma HepG2 cells. Therefore, AEG-1 inhibition was investigated in order to determine whether it was able to downregulate Stat3 phosphorylation. Stat3 activation was observed to be suppressed in the HepG2 cells with shAEG-1 plasmid transfection, compared with that in the empty vector-transfected cells, which was confirmed by reduced tyrosine (Y705) phosphorylation of Stat3 (Fig. 4A). Furthermore, the expression of cell survival-related genes that are regulated by Stat3 were identified in the AEG-1-silenced HepG2 cells. Bcl-2 and Cryab mRNA expression levels were reduced in the cells that were transfected with the shAEG-1 plasmid, compared with those in the mice that were transfected with the empty vector (Fig. 4B; P<0.05).

### Knockdown of AEG-1 inhibits the growth of subcutaneous tumors in nude mice

**Figure 5:**
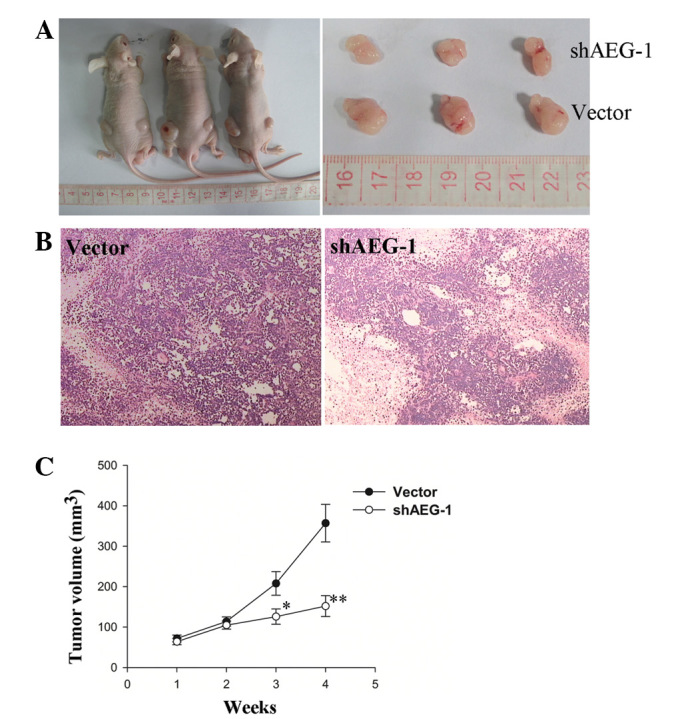
Knockdown of AEG-1 inhibits tumor growth. (A) Typical images of the mice and the tumors that were formed. (B) Hematoxylin and eosin staining of subcutaneous tumors in nude mice (magnification, ×200). (C) Growth curve of tumor volumes (^*^P<0.05 and ^**^P<0.01).

Following subcutaneous cell inoculation in BALB/C nude mice for seven days, the rate of tumor formation of the HepG2-shAEG-1 cells was 62.5% (5/8), whereas the tumor formation rate of the HepG2-vector cells was 75% (6/8). Knockdown of AEG-1 in the HepG2 cells inhibited the growth of subcutaneous tumors and the tumor volumes were smaller than those in the mice that were inoculated with the HepG2-vector cells (Fig. 5A). The average tumor volume and growth rate in the HepG2-shAEG-1 cell inoculation group were markedly reduced compared with the vector control group (Fig. 5C; P<0.05 and P<0.01).

## Discussion

The present study shows that the knockdown of AEG-1 may inhibit cell proliferation and promote apoptosis in the liver cancer HepG2 cell line, and the molecular mechanism may involve the suppression of IL-6 secretion and the inhibition of Stat3 activation.

Despite the fact that AEG-1 was first identified in 2002, studies on AEG-1 have increased gradually in the last five years and have mainly focused on the progression of tumors. Previous studies have suggested that AEG-1 is able to promote invasion and metastasis of hepatocellular carcinoma (7,16,17). However, few studies have investigated the role of AEG-1 in the growth of liver cancer cells. Proliferation and apoptosis are two significant aspects during the tumor growth process (18). The poor prognosis of patients with liver cancer is mainly due to rapid growth and metastasis (2). Therefore, it is necessary to identify the association between tumor-related genes and the growth of liver cancer.

In the present study, the stable HepG2 cells were acquired with AEG-1 silencing. Inhibited proliferation, increased apoptosis and cell cycle arrest were observed in the HepG2-shAEG-1 cells. The tumor microenvironment is now considered to be a significant stimulative factor for tumors. Inflammatory cytokines, the main component of the tumor microenvironment, are closely associated with the development and progression of liver cancer (19). Our previous expression microarray analysis suggested that IL-6 and IL-1β levels were markedly changed in AEG-1-overexpressed and -silenced HCC cells (unpublished data). The subsequent experiments demonstrated that IL-6 secretion was inhibited in AEG-1 knockdown cells, and exogenous IL-6 was able to reverse AEG-1 knockdown-induced anti-proliferation and apoptosis.

The IL-6 family of cytokines, including IL-6, ILF and oncostatin M, are known to be able to activate the Stat3 transcription factor (a downstream target of IL-6 signaling) through autocrine or paracrine pathways. Aberrant Stat3 activation has been reported to exist in numerous human carcinomas (20–22). Upregulated phosphorylation of Stat3 is closely associated with the promotion of growth and inhibition of apoptosis in tumor cells (23,24). IL-6 is able to bind to IL-6R/gp130 and activate the Jak/Stat3 pathway. IL-6 is a downstream regulator of AEG-1 in the HepG2 cells. The phosphorylation of Stat3 was further investigated and it was observed that the activation of Stat3 was reduced in the AEG-1-silenced cells. Furthermore, the cell survival-related genes, Bcl-2 and Cryab, which are regulated by Stat3, were downregulated in the AEG-1-silenced cells. Thus, the inhibition of proliferation and the promotion of apoptosis that was induced by AEG-1 silencing in the HepG2 cells was likely to be mediated by the inactivation of Stat3 and the downregulated expression of Bcl-2 and Cryab. However, whether the molecular mechanism identified in the present study is functional in other liver cancer cells and other tumors requires further study.

In conclusion, the present study has demonstrated that AEG-1 plays a significant role in the proliferation and apoptosis of liver cancer HepG2 cells via downregulated IL-6 secretion and Stat3 activation. Furthermore, AEG-1 knockdown inhibits the tumor growth *in vivo*. Therefore, this study provides an improved understanding of the role of AEG-1 in the growth of liver cancer.
